# Modeling Stimulant and Opioid Co-use in Rats Provided Concurrent Access to Methamphetamine and Fentanyl

**DOI:** 10.3389/fpsyt.2022.814574

**Published:** 2022-02-14

**Authors:** Robert W. Seaman, Chris Lordson, Gregory T. Collins

**Affiliations:** ^1^Department of Pharmacology, The University of Texas Health Science Center at San Antonio, San Antonio, TX, United States; ^2^South Texas Veterans Health Care System, San Antonio, TX, United States

**Keywords:** concurrent access, self-administration, methamphetamine, fentanyl, polysubstance use

## Abstract

Concurrent use of stimulants (e.g., methamphetamine) and opioids (e.g., fentanyl) has become increasingly common in recent years and continues to pose an enormous health burden, worldwide. Despite the prevalence, relatively little is known about interactions between the reinforcing effects of stimulants and opioids in this pattern of polysubstance use. The goals of the current study were to evaluate the relative reinforcing and relapse-related effects of methamphetamine and fentanyl using a concurrent access, drug-vs.-drug choice procedure. Male Sprague-Dawley rats were first allowed to acquire self-administration for either 0.1 mg/kg/infusion methamphetamine or 0.0032 mg/kg/infusion fentanyl, independently, after which concurrent access to both drugs was provided. When training doses of methamphetamine and fentanyl were concurrently available, a subset of rats self-administered both drugs, either within a session or alternating across sessions, whereas the remaining rats responded exclusively for one drug. When the cost of the preferred drug was increased (i.e., unit dose reduced), or the cost of the non-preferred drug was decreased (i.e., unit dose increased), choice was largely allocated toward the cheaper alternative. Following extinction of responding, methamphetamine- and fentanyl-paired cues reinstated responding on both levers. Responding reinstated by a priming injection of methamphetamine or fentanyl allocated more responding to the lever previously reinforced by the priming drug. The current studies suggest that choice of methamphetamine and fentanyl is largely allocated to the cheaper alternative, although more co-use was observed than would be expected for economic substitutes. Moreover, they lay the groundwork for more fully evaluating interactions between commonly co-abused drugs (e.g., stimulants and opioids) in order to better understand the determinants of polysubstance use and develop effective treatment strategies for individuals suffering from a polysubstance use disorder.

## Introduction

In the United States alone, substance use has an estimated economic burden of $600 billion annually, and directly contributed to more than 90,000 deaths by overdose in 2020 (1–3). Adding to the complexities of understanding the pathology and developing effective treatment strategies is the increasing awareness that most individuals with a substance use disorder use more than one substance; thus, recent trends suggest the United States is in the midst of an epidemic of polysubstance use [for review, see (4–6)]. Although the co-use of stimulants and opioids has historically involved mixtures of cocaine and heroin, recently there has been a particularly alarming rise in the incidence of methamphetamine and opioid co-use and overdose (7–14). Users have reported a wide variety of reasons for using stimulants and opioids either concurrently or sequentially, including enhanced euphoria of the drug mixture relative to each constituent, use of methamphetamine to alleviate opioid withdrawal symptoms, and as tools to endure homelessness (9, 12). Importantly, the co-use of stimulants and opioids is also associated with much poorer treatment outcomes (e.g., relapse, overdose) (15, 16). Despite this sharp rise in the co-use of methamphetamine and opioids, relatively little is known about interactions between the abuse-related effects of these drugs in either clinical or preclinical settings.

Given the recent increase in problems associated with the concurrent use of stimulants and opioids, it is vitally important to gain a better understanding of the factors that drive this pattern of co-use in order to develop more effective strategies for treating individuals with a polysubstance use disorder. Indeed, although the co-injection of cocaine and heroin (i.e., “speedballs”) has been common for decades (17), recent estimates suggests that the popularity of stimulant-opioid mixtures is growing, with over 50% of treatment-seeking opioid users reporting regular stimulant use (18, 19). In preclinical models, self-administration of mixtures of cocaine and heroin has been demonstrated to produce synergistic increases in extracellular dopamine levels in rats (20). Consistent with this finding are studies in both rodents (21–24) and non-human primates (25–30); but see (31) demonstrating that the reinforcing effects of cocaine and heroin mixtures are similar to, or greater than the reinforcing effects of either constituent alone. Although less is known about interactions between methamphetamine and opioids, evidence suggests that mixtures of methamphetamine and opioids can result in a more robust locomotor stimulation, and enhancements in the reinforcing effects of small, but not large, doses of methamphetamine (32, 33).

Although mixtures studies are appropriate to model the co-use of stimulant and opioid preparations (e.g., “speedballs,” “goofballs”), other approaches are needed to model situations in which the pattern of polysubstance use involves the co-use of stimulants and opioids as independent entities. One powerful method to evaluate interactions between the reinforcing effects of co-abused drugs is to provide subjects concurrent access to both drugs (34). By manipulating the “cost” of the two drugs (e.g., changing the ratio requirement or the unit dose of drug available), it is possible to determine the nature of their interaction in economic terms (i.e., substitutes, complements, or independents) (35, 36). As the cost of one drug is increased, intake of the fixed cost alternative drug may increase (substitutes), decrease (complements), or stay the same (independents). Previous work from our laboratory used a concurrent access procedure in rats to characterize interactions between the reinforcing effects of two stimulant drugs, 3,4-methylenedioxypyrovalerone (MDPV) and cocaine (37). When functionally equivalent doses of MDPV and cocaine (as determined by a progressive ratio schedule of reinforcement) were made concurrently available, responding tended to be allocated toward one lever or the other, with the behavior of a subset of rats maintained almost exclusively by MDPV, whereas for the remaining rats behavior was maintained almost exclusively by cocaine. However, when the “cost” of the preferred drug was increased (or decreased) by altering the unit doses available for self-administration, all rats exclusively allocated their responding toward the cheaper alternative, suggesting that MDPV and cocaine function as economic substitutes. Although similar methods have been used in non-human primates to suggest that cocaine and the ultra-short acting mu-opioid receptor agonist, remifentanil, function as economic substitutes, the extent to which these relationships extend to methamphetamine and fentanyl is unknown (38, 39).

In addition to better understanding interactions between the reinforcing effects of stimulants and opioids, the high rates of relapse, particularly in individuals with a polysubstance use disorder, highlights the urgent need to better understand the factors contributing to drug-seeking/relapse in polysubstance using populations. For instance, although pharmacotherapies exist to treat opioid use disorder, they are largely ineffective at altering cocaine or methamphetamine use, which can in turn promote relapse to opioid use and increase the likelihood of overdose (40–42). In preclinical assays thought to model some aspects of relapse (e.g., drug-primed reinstatement), the capacity of a drug to reinstate responding is often determined by the degree to which it shares discriminative stimulus properties with the previously self-administered drug (e.g., methamphetamine reinstating responding for cocaine, caffeine reinstating responding for MDPV) (43–45). Consistent with this notion, we have recently established a concurrent reinstatement procedure to show that intravenous primes with cocaine, MDPV, or methamphetamine all reinstate comparable levels of responding on levers previously reinforced by MDPV and cocaine, whereas a priming injection of heroin failed to reinstate responding on either lever. Although this suggests that like begets like, it is unclear how histories of concurrent self-administration of drugs from different pharmacological classes would impact the patterns of cue-induced or drug-primed reinstatement. For instance, a history of concurrent use of stimulants and opioids might erode the specificity typically associated with drug-primed reinstatement, and instead expand the spectrum of drugs that will reinstate responding (e.g., opioids will now effectively reinstate responding for stimulants, and vice versa).

The current studies begin to address these gaps in knowledge by establishing a concurrent access self-administration procedure in which rats have access to both methamphetamine and fentanyl in order to address the following hypotheses: (1) concurrent access to a stimulant and an opioid will result in both drugs maintaining responding, rather than the exclusive patterns of responding observed when two stimulants were available; (2) when the available dose of one drug is increased (cost reduced) or decreased (cost increased), responding will be largely re-allocated toward the lever reinforced by the cheaper alternative, although choice is not expected to be exclusive (i.e., methamphetamine and fentanyl will act as imperfect substitutes); and (3) although methamphetamine and fentanyl will reinstate more responding on the levers that they previously reinforced, methamphetamine will also reinstate responding for fentanyl, and fentanyl will also reinstate responding for methamphetamine, albeit at lower levels.

## Methods

### Subjects

Fifteen male Sprague-Dawley rats (275–300 g upon arrival) were purchased from Envigo (Indianapolis, IN, USA) and maintained in a temperature- and humidity-controlled vivarium. Rats were individually housed and maintained on a 14/10-h light/dark cycle (lights on at 6:00 a.m.). All experiments were conducted during the light cycle and sessions were conducted at approximately the same time each afternoon. Rats were provided ad libitum access to Purina rat chow and water except during experimental sessions. All procedures were conducted in accordance with Institutional Animal Care and Use Committee at the University of Texas Health Science Center at San Antonio and the Guide for Care and Use of Laboratory Animals (46).

### Surgery

Rats were anesthetized with 2–3% isoflurane and prepared with chronic indwelling catheters in the left and right femoral veins using procedures similar to those described previously (37, 47, 48). Catheters were tunneled under the skin and attached to a vascular access button placed in the mid-scapular region. Immediately following surgery, rats were administered Penicillin G (60,000 U/rat) subcutaneously to prevent infection and were allowed 5–7 days to recover. Throughout this recovery period, both catheters were flushed daily with 0.5 ml of heparinized saline (100 U/ml). Thereafter, catheters were flushed daily with 0.2 ml of saline prior to, and 0.5 ml of heparinized saline after the completion of self-administration sessions. Catheter patency was assessed using an intravenous infusion of 5 mg/kg methohexital as needed (e.g., an increase in pressure when flushing, extinction of responding). Three rats were unresponsive to methohexital prior to dose manipulation experiments and were excluded from subsequent experiments.

### Drugs

Fentanyl was provided by the National Institute on Drug Abuse Drug Supply Program (Bethesda, MD). D-methamphetamine and ketamine were purchased from Sigma-Aldrich (St. Louis, MO, USA) and Henry Schein (Dublin, OH, USA), respectively. All drugs were dissolved in sterile 0.9% saline and administered intravenously in a volume of 0.1 ml/kg (for self-administration) or 1 ml/kg (for reinstatement tests) based on body weight. Additionally, methohexital was generously provided by Eli Lilly and Company (Indianapolis, Indiana, USA), dissolved in sterile 0.9% saline and administered in a volume of 1.0 ml/kg to check for catheter patency.

### Apparatus

All experiments were conducted in standard operant conditioning chambers located within ventilated, sound-attenuating enclosures (Med Associates, Inc., St. Albans, VT). Each chamber was equipped with two response levers located 6.8 cm above the grid floor and 1.3 cm from the right or left wall. Visual stimuli were provided by two sets of green, yellow, and red LEDs, one set located above each of the two levers, and a white house light located at the top center of the opposite wall. Drug solutions were delivered by variable speed syringe pumps through Tygon tubing connected to a dual channel stainless-steel fluid swivel and spring tether, which was held in place by a counterbalanced arm. Experimental events were controlled, and data were collected using MED-PC IV software and a PC-compatible interface (Med Associates, Inc.).

### Self-Administration

#### Acquisition

Behavior was initially maintained by either 0.1 mg/kg/infusion of methamphetamine or 0.0032 mg/kg/infusion of fentanyl under a fixed ratio (FR) 1: timeout (TO) 5-s schedule of reinforcement during daily 90-min sessions. Doses were chosen based on their relative positions (peak) on their respective progressive ratio dose-response curves (47, 48). Two sets of conditioned stimuli (discriminative and infusion-paired) were used in these studies. The discriminative stimuli paired with methamphetamine and fentanyl were counterbalanced across rats and different for each drug. One discriminative stimulus consisted of the illumination of a yellow LED above the active lever (left or right; counterbalanced across rats) that signaled drug availability. Completion of the response requirement on this lever resulted in a drug infusion (0.1 ml/kg over ~1 s) that was paired with the illumination of the yellow, green, and red LEDs above that lever as well as the houselight; these lights remained illuminated for the duration of the 5-s post-infusion timeout period during which no additional infusions could be earned. The other set of discriminative stimuli consisted of the illumination of green and red LEDs above the active lever (left or right; counterbalanced across rats) that signaled drug availability. Completion of the response requirement on this lever resulted in a drug infusion (0.1 ml/kg over ~1 s) that was paired with the flashing of the yellow, green, and red LEDs as well as the houselight, at 1 hz; this occurred throughout the 5-s post-infusion timeout period during which no additional infusions could be earned. Responses made on the inactive lever, and those made on either lever during timeouts, were recorded but had no scheduled consequences. Acquisition criteria were defined as: ≥12 infusions for two consecutive days with ≥80% responding occurring on the active relative to inactive lever. Response requirements were subsequently increased to an FR 5 where they remained for the duration of the study. After 7 days, and once behavior met stability criteria for the initial drug (±20% of the mean of two consecutive sessions), behavior was now maintained by the alternate drug on the alternate lever (and alternate set of conditioned stimuli) under an FR 5 schedule. The initially active lever now became inactive (i.e., the discriminative stimuli were omitted and responding had no programmed consequences). This condition was kept in place for at least 10 sessions and until stability criteria were met to allow for nearly equal exposure to both drugs prior to being provided concurrent access. Throughout the entire acquisition period (i.e., acquisition of responding for both methamphetamine and fentanyl), the catheter through which drug infusions were delivered alternated daily in order to ensure that both catheters functioned equivalently.

#### Concurrent Access

After reaching stability under an FR 5 schedule for the second drug, access to both drugs (or saline) was provided and their associated stimuli under a concurrent FR5:FR5 schedule of reinforcement during daily 90-min sessions. For all rats, the following conditions were evaluated in quasi-random order: (1) concurrent access to 0.1 mg/kg/infusion of methamphetamine and saline; (2) concurrent access to 0.0032 mg/kg/infusion of fentanyl and saline; and (3) concurrent access to 0.1 mg/kg/infusion of methamphetamine and 0.0032 mg/kg/infusion of fentanyl. Conditions remained in place for 7 sessions. Each session began with a 1-min blackout followed by two sample trials, one on each lever, for the available drug (or saline) and stimulus conditions. A 1-min blackout followed each sample trial. The order of sample trials (i.e., drug and stimuli) was counter-balanced across rats. The session counter did not begin until 1 min after the second sample trial was completed. Throughout the remainder of the session, rats had concurrent access to both drugs (or one drug and saline) and associated stimuli.

#### Dose-Substitution

Subsequent to establishing preference between training doses of methamphetamine and fentanyl, the following manipulations were made in order to evaluate economic interactions between methamphetamine and fentanyl: (1) the unit dose of the more preferred drug was decreased by ½ log (i.e., cost increased); and (2) the unit dose of the less preferred drug was increased by ½ log (i.e., cost decreased). For instance, if a rat self-administered more of methamphetamine (0.1 mg/kg/infusion) than fentanyl (0.0032 mg/kg/infusion), the unit dose of methamphetamine was decreased (0.032 mg/kg/infusion methamphetamine vs. 0.0032 mg/kg/infusion fentanyl) or the unit dose of fentanyl was increased (0.1 mg/kg/infusion methamphetamine vs. 0.01 mg/kg/infusion fentanyl). The order of these dose manipulations was quasi-random, with each condition maintained for 7 sessions.

#### Extinction and Reinstatement

Upon completion of the dose manipulation studies, responding on both levers was extinguished and a series of reinstatement tests were conducted in order to determine the pattern of reinstatement behavior in rats with a history of concurrent access to methamphetamine and fentanyl. These tests included: (1) reintroduction of both the methamphetamine- and fentanyl-associated stimuli (cue-induced reinstatement); and (2) drug primes with methamphetamine (0.32 mg/kg; IV), fentanyl (0.032 mg/kg; IV), or ketamine (3.2 mg/kg; IV), administered 5 min before the start of a test session. Briefly, under extinction conditions, discriminative stimuli for both drugs were omitted and completion of response requirements on either lever had no programmed consequences (i.e., no infusions or infusion-paired stimuli were delivered). Extinction conditions remained in place for at least 7 sessions, and until the total number of lever responses on both levers was ≤ 15% of baseline responding. Once extinction criteria were met, a series of 4 reinstatement tests were performed as described previously (37, 44). Briefly, reinstatement tests were identical to self-administration conditions with the exceptions that: (1) intravenous pretreatments of saline (cue-induced reinstatement) or drug (cue + drug-primed reinstatement) were administered 5 min before the session; (2) sample trials were omitted from the session; and (3) completion of response requirements resulted in the delivery of a saline infusion in conjunction with the methamphetamine- or fentanyl-associated stimuli. Both sets of discriminative stimuli and conditioned stimuli were present in all reinstatement tests. Cue-induced reinstatement always occurred first followed by three additional cue + drug-primed reinstatement tests. Cue + drug-primed tests occurred in a quasi-random order, with each reinstatement test separated by at least two extinction sessions; additional extinction sessions were conducted until the extinction criterion was met.

### Data Analysis

All data are presented as the mean ± S.E.M. For dose-substitution studies, the percent choice of 0.1 mg/kg/infusion of methamphetamine is shown as a function of fentanyl dose (or saline) whereas the percent choice of 0.0032 mg/kg/infusion of fentanyl is shown as a function of methamphetamine dose (or saline). Data represent the average of the final three sessions of each dose-substitution period and were analyzed *via* a mixed-effects repeated measure one-way analysis of variance (ANOVA) and *post-hoc* Dunnett's test comparing the percent drug choice at each dose available vs. when saline is available. Extinction data were analyzed via a two-way repeated measure ANOVA (factors being time and lever) and *post-hoc* Dunnett's test comparing the number of responses on each lever relative to the first day of extinction. Similarly, data from reinstatement tests were analyzed via a mixed-effects two-way repeated measure ANOVA (factors being pretreatment and lever) and *post-hoc* Dunnett's test when comparing responding on each lever to extinction responding, and Bonferroni's test when comparing allocation of responding on each lever produced by each pretreatment.

## Results

### Acquisition and Single-Drug Access

**Figure 1:**
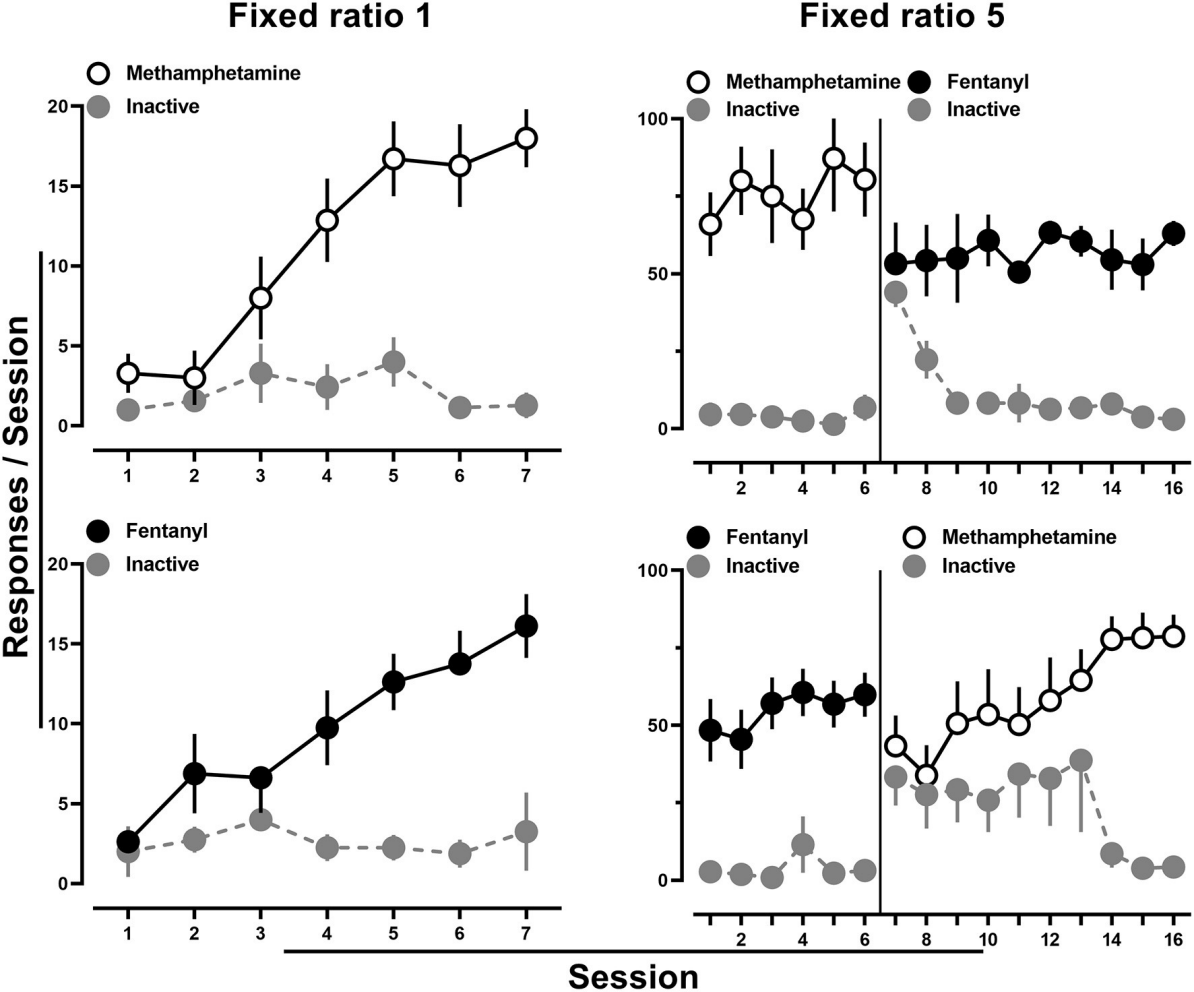
Number of active and inactive lever responses throughout the 7-day acquisition period for methamphetamine (0.1 mg/kg/infusion) (upper left) or fentanyl (0.0032 mg/kg/infusion) (lower left). Subsequent responding under a fixed ratio five schedule of reinforcement for methamphetamine and when fentanyl was substituted on the previously inactive lever (upper right). Similarly, responding under a fixed ratio five schedule of reinforcement for fentanyl followed by methamphetamine substitution on the previously inactive lever (lower right). The solid line represents when the alternate drug and drug-paired stimuli were made available on the alternate lever. Data represent the mean ± S.E.M., and each point represents 7–8 rats.

All rats provided access to methamphetamine (0.1 mg/kg/infusion) met acquisition criteria by the 7th session (Figure 1; upper left), and methamphetamine intake was maintained upon increasing the fixed ratio to 5 (Figure 1; upper right). When fentanyl (0.0032 mg/kg/infusion) was then introduced and made available for responding on the previously inactive lever, rats readily reallocated their responding to this lever, with nearly exclusive responding on the now fentanyl-reinforced lever observed by the end of 10 sessions (Figure 1; upper right). Similarly, acquisition criteria were met in all rats provided access to fentanyl (0.0032 mg/kg/infusion) (Figure 1; lower left), and intake was maintained upon increasing the fixed ratio to 5 (Figure 1; lower right). When methamphetamine (0.1 mg/kg/infusion) was next introduced and made available for responding on the previously inactive lever, rats readily reallocated responding to this lever, with nearly exclusive responding on the methamphetamine lever observed by the end of 10 sessions (Figure 1; lower right). Throughout this period, there were no apparent differences in drug intake as a function of the catheter through which drug was infused.

### Concurrent Access

**Figure 2:**
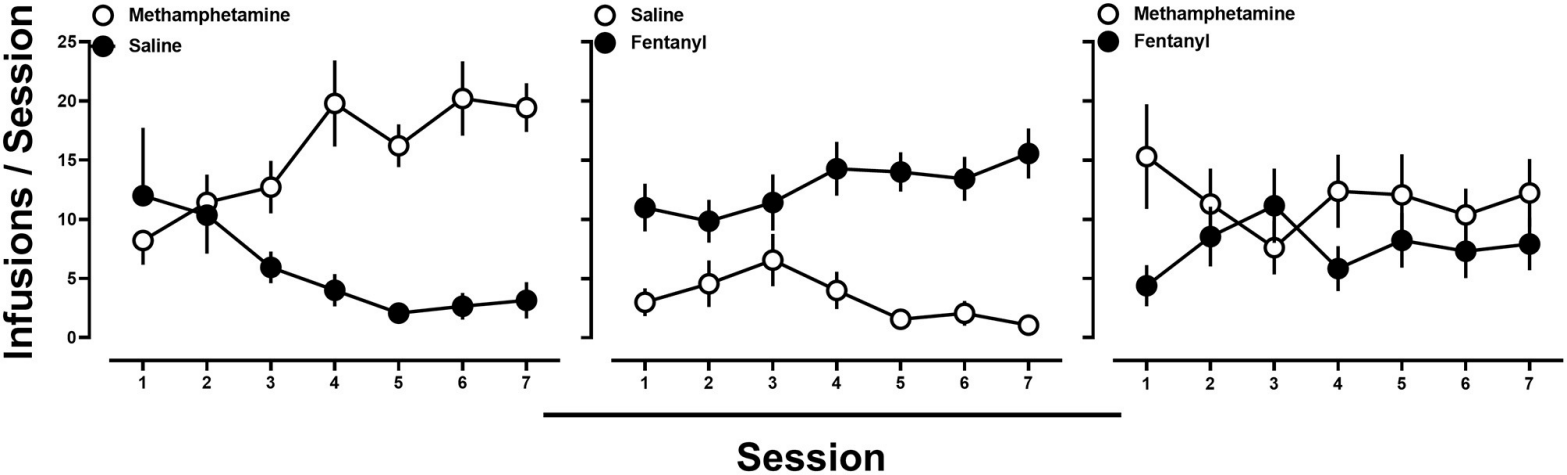
**(Left)** Average number of infusions of 0.1 mg/kg/infusion methamphetamine (open circles) and saline (filled circles) when methamphetamine and saline were concurrently available. **(Middle)** Average number of infusions of 0.0032 mg/kg/infusion fentanyl (filled circles) and saline (open circles) when fentanyl and saline were concurrently available. **(Right)** Average number of infusions of 0.1 mg/kg/infusion of methamphetamine (open circles) and 0.0032 mg/kg/infusion of fentanyl (filled circles) when methamphetamine and fentanyl were concurrently available. Data represent the mean ± S.E.M., and each point represents 14–15 rats.

**Figure 3:**
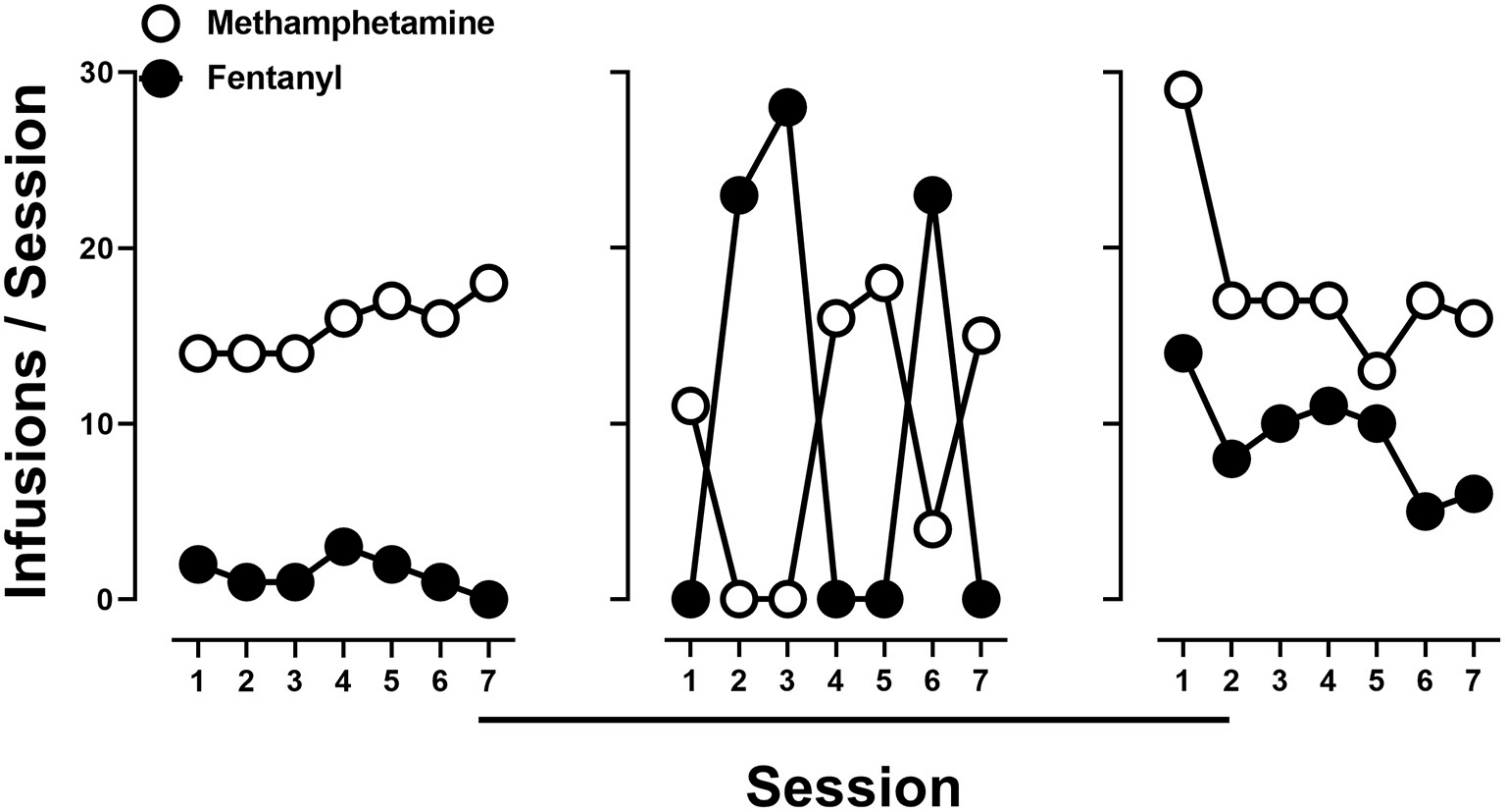
Number of infusions of 0.1 mg/kg/infusion of methamphetamine (open circles) and 0.0032 mg/kg/infusion of fentanyl (filled circles) when methamphetamine and fentanyl were concurrently available in representative rats demonstrating different patterns of drug intake.

Subsequently, rats were provided access to methamphetamine (0.1 mg/kg/infusion) and saline, fentanyl (0.0032 mg/kg/infusion) and saline, or methamphetamine (0.1 mg/kg/infusion) and fentanyl (0.0032 mg/kg/infusion), in a pseudorandom order. When methamphetamine and saline (Figure 2; left) or fentanyl and saline (Figure 2; middle) were concurrently available, responding was nearly exclusively allocated toward the lever that was reinforced by drug by the end of the 7 sessions. In contrast, when the training doses of methamphetamine and fentanyl were available concurrently, responding, at the group level, occurred at comparable levels on both the methamphetamine- and fentanyl-reinforced levers (Figure 2; right). Upon examination of individual subject data, three general patterns of responding were observed. One group (*n* = 6) of rats tended to respond nearly exclusively for either methamphetamine (*n* = 2) or fentanyl (*n* = 4) over the course of the seven sessions (Figure 3; left; representative rat), whereas another subset of rats (*n* = 3) tended to exhibit exclusive responding for one drug, but preference for methamphetamine or fentanyl alternated across days (Figure 3; middle; representative rat), and the remaining rats (*n* = 5) consistently responding for both methamphetamine and fentanyl across each of the seven sessions (Figure 3; right; representative rat).

### Dose Substitution

**Figure 4:**
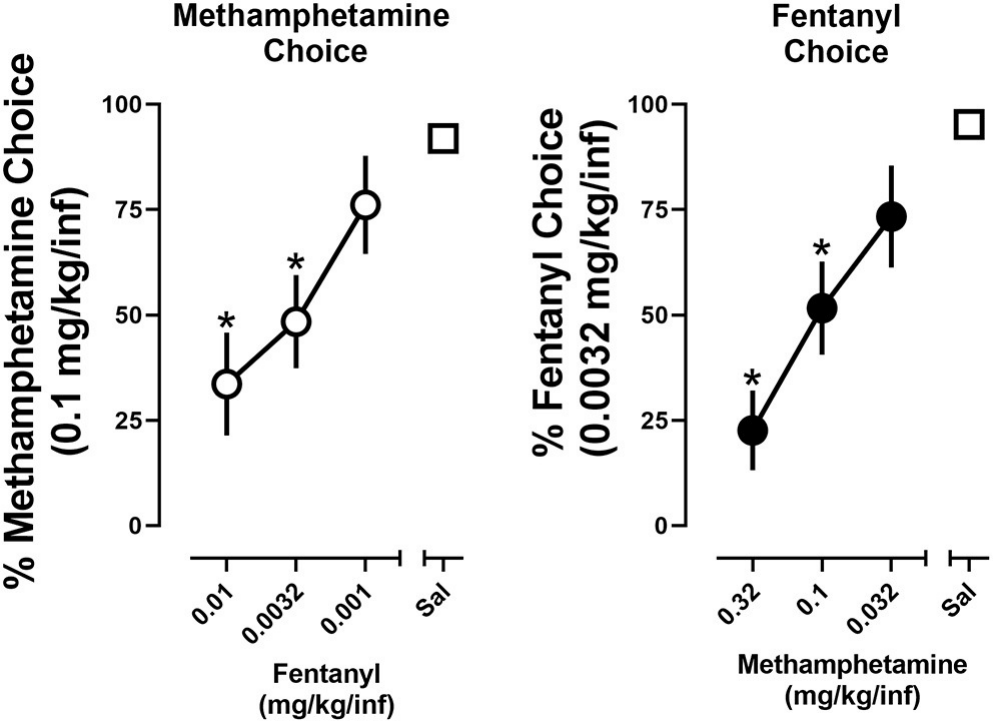
Percent choice of 0.1 mg/kg/infusion of methamphetamine as a function of concurrently available fentanyl dose (or saline) **(left)**. Percent choice of 0.0032 mg/kg/infusion of fentanyl as a function of concurrently available methamphetamine dose (or saline) **(right)**. Data represent the mean ±S.E.M. Each point represents 8–12 rats. Asterisks represent a significant decrease from saline (*p* < 0.05).

To evaluate economic interactions between methamphetamine and fentanyl, the cost of one drug was either increased (unit dose decreased) or decreased (unit dose increased) while the cost of the alternative drug remained fixed. When the cost of methamphetamine remained constant, choice of methamphetamine increased as the cost of fentanyl increased (i.e., rats chose 0.1 mg/kg/infusion methamphetamine over 0.001 mg/kg/infusion fentanyl) (Figure 4; left). A significant effect of dose [*F*_(2, 17.7)_ = 11.2; *p* < 0.0001] was revealed by a one-way repeated measure ANOVA, with *post-hoc* tests indicating that choice of methamphetamine was significantly reduced when either 0.0032 mg/kg/infusion (48.4%) or 0.01 mg/kg/infusion (33.6%) of fentanyl was made concurrently available, as compared to when methamphetamine and saline were concurrently available (91.9%). Similarly, when the cost of fentanyl remained constant (FR5 for 0.0032 mg/kg/infusion), choice of fentanyl increased as the cost of methamphetamine increased (i.e., rats chose 0.0032 mg/kg/infusion fentanyl over 0.032 mg/kg/infusion methamphetamine) (Figure 4; right). A significant effect of dose [*F*_(2.5, 21.5)_ = 12.4; *p* < 0.0001] was revealed by a one-way repeated measure ANOVA, with *post-hoc* tests indicating that choice of fentanyl was significant reduced when either 0.1 mg/kg/infusion (51.6%) or 0.32 mg/kg/infusion (22.6%) of methamphetamine was made concurrently available, as compared to when fentanyl and saline were concurrently available (95.2%).

### Extinction and Reinstatement

**Figure 5:**
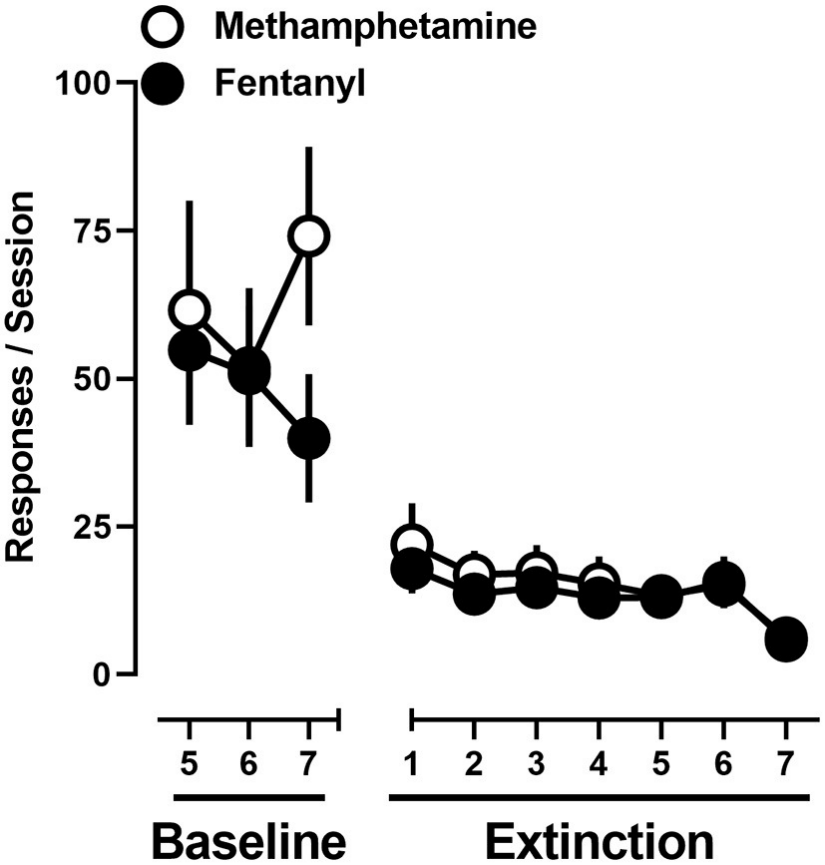
Responses made on the methamphetamine (open circles) and fentanyl (filled circles) levers on the final 3 days of concurrent access to methamphetamine and fentanyl self-administration, and extinction conditions. Data represent the mean ± S.E.M., and each point represents 12 rats.

Under baseline conditions in which rats were provided concurrent access to 0.1 mg/kg/infusion methamphetamine and 0.0032 mg/kg/infusion of fentanyl, responding, at the group level, was allocated toward both levers. Upon instituting extinction conditions, responding on levers previously reinforced by methamphetamine or fentanyl decreased across sessions with extinction criteria met on day 6 ± 0.8. A two-way repeated-measure ANOVA revealed that there was no significant difference in extinction of responding on the methamphetamine and fentanyl levers [*F*_(1, 11)_ = 0.26; *p* > 0.05], nor a main effect of time [*F*_(2.3, 24.9)_ = 2.8; *p* = 0.08] (Figure 5).

**Figure 6:**
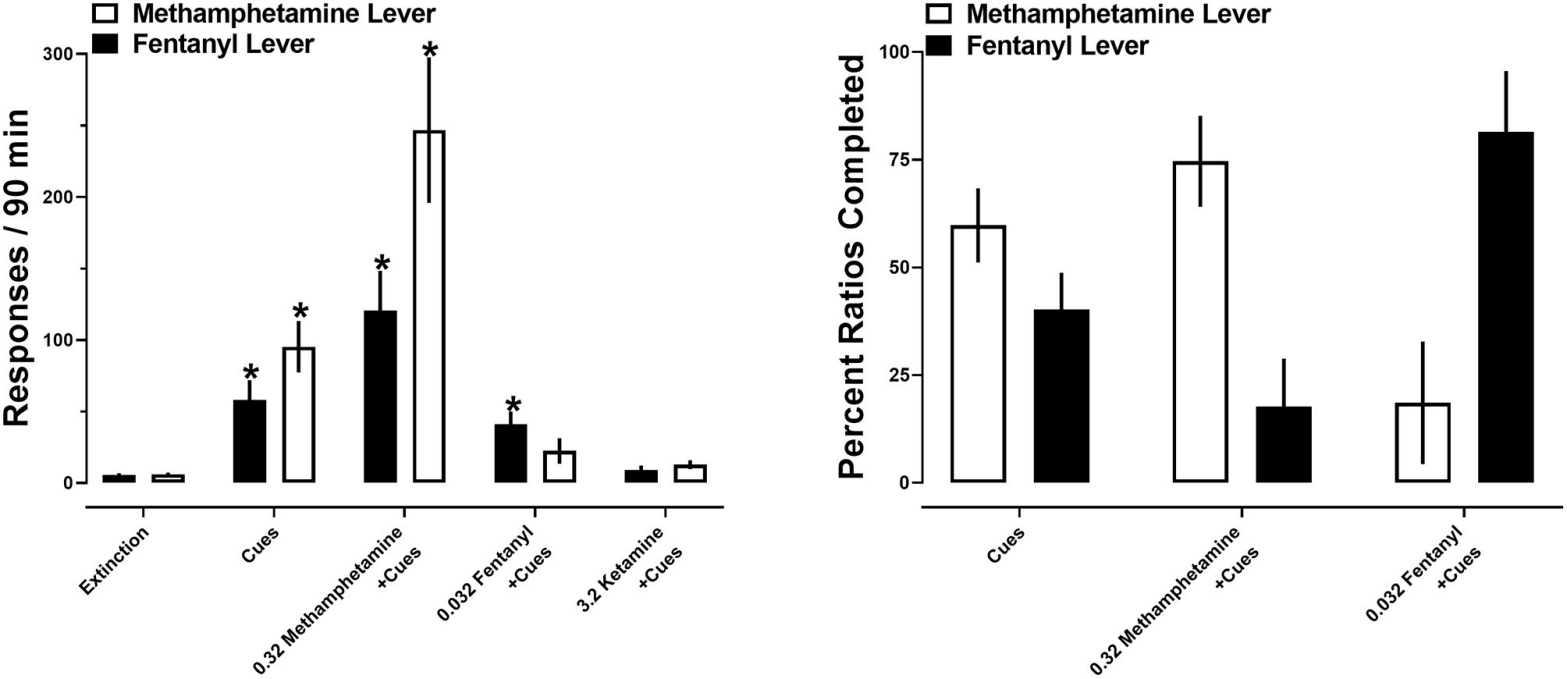
**(Left)** Responses made on the methamphetamine (open bars) and fentanyl (closed bars) levers on the final day of extinction and during reinstatement tests. Asterisks represent a significant increase from extinction (*p* < 0.05). Each bar represents 12 rats. **(Right)** percent of the first 10 ratios completed on the methamphetamine (open bars) and fentanyl (closed bars) levers during reinstatement tests. Each set of bars represents 8–12 rats. All data represent the mean ± S.E.M.

After extinction criteria were met, a series of reinstatement tests were conducted. Reintroduction of drug-paired cues produced 95 ± 18 responses on the methamphetamine lever and 58 ± 14 responses on the fentanyl lever. When drug-paired cues were reintroduced in conjunction with a priming injection of methamphetamine, a greater number of responses occurred on the methamphetamine lever (247 ± 51) relative to the fentanyl lever (120 ± 28). The opposite was true when a priming injection of fentanyl was administered, with more responding being produced on the fentanyl lever (41 ± 9) than the methamphetamine lever (22 ± 9). Ketamine produced the fewest number of responses, with 13 ± 3 and 9 ± 3 responses being made on the methamphetamine and fentanyl levers, respectively (Figure 6; left).

A two-way repeated measures ANOVA revealed no significant main effect of lever [*F*_(1, 11)_ = 4; *p* > 0.05], but a significant main effect of drug primes [*F*_(1.3, 14.1)_ = 29.3; *p* < 0.0001] and a significant interaction between lever and drug primes [*F*_(1.2, 13.2)_ = 5.5; *p* < 0.05]. *Post-hoc* analyses indicated that reintroduction of drug-paired cues significantly increased responding on both the methamphetamine and fentanyl levers (*p* < 0.05), relative to the final day of extinction. Similarly, drug-paired cues in conjunction with a prime with methamphetamine significantly increased responding on both the methamphetamine and fentanyl levers (*p* < 0.05), relative to the final day of extinction. In contrast, a priming injection of fentanyl significantly increased responding on the fentanyl, but not methamphetamine, lever, relative to the final day of extinction. No significant increases in responding were observed following primes with ketamine. When comparing total responding on each lever as a function of pretreatment, there were no significant differences in the number of responses on each lever within each reinstatement test (Figure 6; left).

When analyses were restricted to the first ten ratios completed, reintroduction of drug-paired cues resulted in similar allocation of responding, resulting in 60% of ratios completed on the methamphetamine lever and 40% completed on the fentanyl lever. In contrast, a priming injection of methamphetamine shifted this ratio, resulting in 75% of the first 10 completed ratios completed on the methamphetamine lever, and 25% completed on the fentanyl lever. A priming injection of fentanyl produced more completed ratios on the fentanyl lever (81%) relative to the methamphetamine lever (19%). A two-way repeated measures ANOVA no main effects of lever [*F*_(1, 54)_ = 0.24; *p* > 0.05] or drug primes [*F*_(1, 27)_ = 0.09; *p* > 0.05] (Figure 6; right).

## Discussion

Polysubstance use involving methamphetamine and fentanyl is common within substance using populations, yet little is known about the pharmacological and behavioral factors that drive this growing threat to public health. The current studies established a concurrent access self-administration procedure to model the co-use of methamphetamine and fentanyl in rats and to determine economic interactions between methamphetamine and fentanyl and how a history of concurrent access to both drugs impacts relapse-related behaviors. There were 3 main findings: (1) when rats were provided concurrent access to methamphetamine and fentanyl, responding for methamphetamine and fentanyl was comparable at the group level; however, at the individual subject level different patterns of drug-taking were observed with some rats responding on both reinforced levers whereas others exhibited exclusive choice of one drug; (2) methamphetamine and fentanyl acted as imperfect substitutes, that is to say, when the cost of one drug was increased, responding was largely, but not exclusively, reallocated toward the fixed cost alternative, and when the cost of one drug was decreased responding was largely, but not exclusively, reallocated toward the now cheaper alternative; and (3) reintroduction of the drug-paired cues reinstated responding on both the methamphetamine and fentanyl levers whereas drug-paired cues in conjunction with priming injections of methamphetamine or fentanyl produced responding that was largely allocated toward the levers previously reinforced by methamphetamine or fentanyl, respectively. Taken together, these data suggest that methamphetamine and fentanyl can act as imperfect substitutes and increase the breadth of conditions that produce relapse-related behaviors.

Rats provided concurrent access to methamphetamine and fentanyl exhibited different patterns of intake. Although a subset of rats responded exclusively for methamphetamine or fentanyl across the 7-day testing block, the majority of rats responded for both methamphetamine and fentanyl, albeit in slightly different manners. Some rats alternated exclusive responding for either methamphetamine or fentanyl across days, whereas the remaining rats maintained concurrent methamphetamine and fentanyl intake within each session. The prevalence of rats responding for both methamphetamine and fentanyl in the current studies is in stark contrast to what was observed when rats are provided concurrent access to two drugs from the same class, MDPV and cocaine (37). In those studies, MDPV and cocaine acted as economic substitutes with nearly exclusive choice occurring in all subjects and determined by the relative cost of each drug. Rats oftentimes responding for both methamphetamine and fentanyl in the current studies mirrors reports of human drug users preferring to use stimulant and opioids together rather than in isolation. Indeed, concurrent use of methamphetamine and opioids has been reported to produce an enhanced euphoria or, “high” while circumventing the unwanted side effects of each drug, and aid in forestalling opioid withdrawal (9), suggesting a potentially synergistic interaction between the two drugs. Preclinical models, such as the concurrent access procedure employed herein, capable of elucidating the factors contributing to these different patterns of intake will result in a better understanding of the human condition and ultimately aid in the development of more effective therapeutic strategies for those engaged in polysubstance use.

In addition to simply evaluating patterns of intake, concurrent access procedures allow for the economic analyses of the interactions between co-used drugs (e.g., substitutes, complements, or independents) which can provide additional insights into the reinforcing effects of each drug under situations more closely related to polysubstance use (35, 36). In the current studies, when rats were provided concurrent access to varying intravenous doses of methamphetamine and fentanyl (i.e., the cost of each drug was manipulated in the presence of the training dose of the alternative), more responding was allocated toward the cheaper alternative, however, responding tended not to be exclusive, suggesting that stimulants and opioids appear to function as imperfect substitutes. This is in contrast to the largely exclusive choice that was observed when two drugs of the same class, MDPV and cocaine, were concurrently available (37) and suggests that although cost might largely dictate choice of methamphetamine or fentanyl, there are other contributors to drug choice when a stimulant and opioid are concurrently available (e.g., a possible synergistic interaction between the two drugs). One consideration regarding the interpretation of these data is that for this initial study, varying doses of each drug were evaluated only when the training dose of the other drug was concurrently available. Regardless, methamphetamine and fentanyl acting as substitutes in the current studies support previous work demonstrating poorer treatment outcomes for individuals suffering from polysubstance use disorder (15, 16, 42). For instance, if an individual using stimulants and opioids is effectively treated for their opioid use disorder, but continues to use stimulants, it is possible that the ongoing use of stimulants could increase the likelihood of relapse to opioid-taking, thereby paving the way for a return to regular polysubstance use (15). Although a more thorough evaluation of doses will need to be completed in both male and female subjects in order to more fully define the nature of the economic interactions between methamphetamine and fentanyl, the present data suggest that methamphetamine and fentanyl act as imperfect substitutes, likely contributing to the high prevalence of co-use of these two drugs either together, or in place of one another.

Although available evidence from treatment-seeking individuals suggest that polysubstance use is associated with poorer treatment outcomes, including higher rates of relapse and overdose (15, 16), relatively few preclinical studies have investigated relapse-related behaviors in the context of polysubstance use. In the current studies, reintroduction of drug cues previously associated with concurrent access to methamphetamine and fentanyl reinstated responding on both levers to a similar degree. Although methamphetamine- and fentanyl-primed reinstatement increased responding on both drug-paired levers, more responding was allocated to the lever associated with the priming drug administered. This is consistent with what has been observed in reinstatement studies wherein rats have a history of self-administering cocaine and heroin (49). Analysis of the first ten ratios that were completed in reinstatement tests demonstrated that when drug-paired cues were reintroduced alone, the first ten ratios completed were equally distributed across both methamphetamine and fentanyl levers on the group level, the result of all rats responding on both levers to varying degrees. In contrast, a pretreatment with methamphetamine or fentanyl resulted in a larger number of ratios being completed on the lever associated with methamphetamine or fentanyl, respectively. Our laboratory has recently demonstrated that reintroduction of drug-paired cues alone, as well as in conjunction with primes of MDPV, cocaine, or methamphetamine, produced responding on both drug paired levers in rats with a history of concurrent MDPV and cocaine self-administration, with more responding generally occurring on the cocaine-paired lever, regardless of priming drug or drug preference (37). Analyses of the first ten ratios completed during reinstatement tests reveal subtle differences in reinstatement behavior when drugs previously self-administered belong to the same class, or different classes. The initial ratios completed in MDPV- or cocaine-primed reinstatement tests in subjects having a history of concurrent MDPV and cocaine self-administration were largely allocated toward the previously reinforced cocaine lever, regardless of which drug was administered or the drug preference of a given subject. However, the current studies demonstrate that methamphetamine- or fentanyl-primed reinstatement results in the initial ten ratios largely being completed on the lever associated with the priming drug, in subjects having a history of concurrent methamphetamine and fentanyl self-administration. Importantly, in the current studies, a drug with non-overlapping discriminative stimulus effects with methamphetamine or fentanyl, in this case ketamine, did not increase responding greater than that produced by cues alone. This is not altogether surprising given the concordance between drug discrimination and drug-primed reinstatement. Indeed, in rats trained to discriminate two drugs on different operanda, administration of a compound producing non-overlapping discriminative stimuli with either training drug can result in a lack of responding (50–52). It is also possible that the dose of ketamine was sufficient to suppress responding, however, rats will self-administer this unit dose of ketamine, with total levels of ketamine intake in excess of 40 mg/kg during a 90-min session (53, 54). These findings support a primary role for discriminative stimulus effects in drug-primed reinstatement, but also suggest that a history of concurrent self-administration of drugs from different classes (e.g., methamphetamine and fentanyl) may degrade the specificity of drug-primed reinstatement of responding. Although this notion is supported by the current studies, additional studies are needed to more fully characterize the consequences of co-use of methamphetamine and fentanyl on reinstatement behavior, including the evaluation of a larger range of priming doses, and evaluating reinstatement behavior following priming injections of mixtures of methamphetamine and fentanyl. Taken together, these data suggest that environmental and pharmacological stimuli associated with the use of a particular substance (e.g., a spoon and syringe for heroin, or a glass pipe for methamphetamine) might trigger a more general drug-seeking response in individuals with a history of polysubstance use, rather than a more specific desire to use the substance associated with those stimuli.

Despite the growing awareness that polysubstance use is the norm rather than the exception, the vast majority of preclinical substance use research continues to focus on the effects of individual drugs, studied in isolation. The current studies established a concurrent access self-administration procedure to investigate interactions between the reinforcing effects of methamphetamine and fentanyl and found them to function as imperfect substitutes with at least three different patterns of drug-taking emerging when both drugs were concurrently available. This is in contrast to what is observed when rats are provided concurrent access to two stimulants (37), but consistent with reports from polysubstance users that suggest that concurrent co-use of stimulants and opioids is preferable to the use of either drug alone (9, 12). Although reintroduction of both sets of drug-paired stimuli would be expected to reinstate responding on both the methamphetamine and fentanyl levers, that priming injections of methamphetamine or fentanyl also increased responding on both levers was somewhat unexpected and suggests that environmental and pharmacological stimuli may have a more general, but complex, influence on relapse-related behaviors in polysubstance users. These studies lay the groundwork for a deeper evaluation of the interactions between the reinforcing effects of methamphetamine and fentanyl using drug-vs.-drug choice. For instance, previous studies from our laboratory and others have demonstrated that the reinforcing effects of opioids, but not stimulants, are enhanced when subjects are in a state of opioid withdrawal (47, 55, 56). However, the degree to which opioid withdrawal would impact preference for and/or economic interactions between methamphetamine and fentanyl is an important and underexplored aspect of the current epidemic of polysubstance use.

## Data Availability Statement

The original contributions presented in the study are included in the article/supplementary material, further inquiries can be directed to the corresponding authors.

## Ethics Statement

The animal study was reviewed and approved by Institutional Animal Care and Use Committee at the University of Texas Health Science Center at San Antonio.

## Author Contributions

RS and GC contributed to study design and wrote the manuscript. RS and CL conducted behavioral experiments. RS performed data analysis. All authors critically reviewed content and approved final version for publication.

## Funding

This research was supported by National Institutes of Health and National Institute on Drug Abuse [R01DA039146 (GC)].

## Conflict of Interest

The authors declare that the research was conducted in the absence of any commercial or financial relationships that could be construed as a potential conflict of interest.

## Publisher's Note

All claims expressed in this article are solely those of the authors and do not necessarily represent those of their affiliated organizations, or those of the publisher, the editors and the reviewers. Any product that may be evaluated in this article, or claim that may be made by its manufacturer, is not guaranteed or endorsed by the publisher.
